# Editorial: Multidimensional interplay of early-life events, neuroactive steroids and sex in the development of psychopathology and psychiatric disorders, volume 1

**DOI:** 10.3389/fnbeh.2022.1036055

**Published:** 2022-10-24

**Authors:** Roberto Frau, Fabrizio Sanna, Liana Fattore

**Affiliations:** ^1^Department of Biomedical Sciences, Section of Neurosciences and Clinical Pharmacology, University of Cagliari, Cagliari, Italy; ^2^The Guy Everett Laboratory for Neuroscience, University of Cagliari, Cagliari, Italy; ^3^CNR Institute of Neuroscience-Cagliari, National Research Council, Cagliari, Italy

**Keywords:** neuroactive steroids, neurosteroids, sex difference, sex hormones, neurodevelopment, neuropsychiatric disorders, neuropharmacology, environmental risk factors

The prevalence of psychiatric disorders has dramatically increased and remained among the top 10 leading causes of disease burden worldwide Global Burden of Disease, (GBD, 2019). Between 1990 and 2019, their global prevalence rose by nearly 50%, and, despite decades of research, psychiatric disorders still remain the diseases with the lowest probability of success. Apart from very few exceptions, over the last years, no new drug targets have been identified and almost all the recently introduced molecules are based on revised engineering of the mechanisms of benchmark drugs. The reasons for this failure are multi-factorial, but they all converge on the lack of a full understanding of the etiology and pathophysiology of psychiatric disorders.

The last decade, however, has witnessed a significant advance in the knowledge of mental diseases, especially because we have substantially changed our thinking of and approach to psychiatric disorders. We have gained new awareness: the influence of genes manifests earlier than expected and their expression and differentiation into discrete psychiatric syndromes require the presence of additional interacting factors (e.g., social, environmental), which are capable of shaping the phenotypic expression upon alterations in brain circuitry and function (Cross-Disorder Group of the Psychiatric Genomics Consortium, 2019). In the complex interplay between genetic makeup and environmental risk factors, the biological factor of sex is receiving increasing attention as a key determinant in the susceptibility to develop psychopathologies. More and more scientists are now systemically including sex-related variables in experimental studies to unravel the influence of sex (and its hormonal components) on the development of psychiatric diseases. Moreover, we implemented animal models based on etiological validities (homologies between the etiologies in the animal model and the psychiatric condition) and “multiple hits” hypotheses, where stress, prenatal exposure to drugs of abuse and infections were the most-used environmental risk factors able to unveil a psychopathological phenotype.

This Special Issue faces the multi-factorial origin of psychiatric disorders, taking into account the contribution of multiple environmental and biological risk factors, such as sex, use of addictive substances, early stress, and prenatal insults. The challenge is to approach mental illness with strategies based on early prevention and intervention as well as disease-modification, overcoming the unsatisfactory categorical diagnosis used in psychiatric practice. The original studies and comprehensive reviews collected here contribute to clarifying some of the neurobiological underpinnings imposed by risk factors on brain function and behavior, embracing preventive strategies to curb the social and medical burden imposed by psychiatric disorders.

An increasing body of knowledge is pointing out the crucial role played by Transposable Elements (TEs) across evolution (from environment adaptation to promotion of eusociality) and in several regulatory functions in mammals, which have long been considered junk DNA or DNA parasites, although for most mammals they represent up to 50% of their genome. The elegant review by Chiang et al. provides an up-to-date view of the recent advances in understanding the roles of TEs in the complex interplay between gonadal steroids and epigenetic processes in sexual development and differentiation. Unique sex-specific vulnerabilities have also been detected in the consequences of prenatal drug exposure on the brain and behavior of newborns during infancy, adolescence, and adulthood. Thus, as the second contribution of this collection, the mini-review by Sikic et al. revisits clinical and pre-clinical studies on prenatal nicotine exposure (PNE) and discusses the modulating role of sex in the manifestation of PNE-related outcomes. Since the methodology used in the different studies resulted in outcomes that were too inconsistent to define the role of sex in this context, the authors recommended the inclusion of sex as a discriminating factor in PNE research along with the increased use of vapor exposure models in preclinical research to more accurately model the parameters of human nicotine intake. Another important aspect of the interplay between early-life events, sex, and the risk to develop a psychiatric disorder is the availability of the mother to provide optimal care for her offspring, since limited environmental resources may result in atypical maternal care. The study by Kent et al. explored the mechanisms associated with adaptive and maladaptive behavioral responses to disruptions in physical and social postnatal contexts in male and female rats born from mothers housed in standard or limited nesting resources. The authors show how a reduction in material resources during postnatal development results in sex-dependent alterations not only in play behavior, exploration strategies, and cognition but also in morphometric and musculoskeletal ones. Due to the dense interconnections between the bones and brain, including the endocrine functions of the skeletal system, this study highlights the importance to consider the reciprocal interactions between the nervous system and other physiological systems (i.e., the musculoskeletal system) when investigating adaptive responses to environmental factors.

Adolescence is a critical period of development due to highly impacting changes taking place both in the brain and the body, with interacting physiological processes promoting the completion of sexual development (under the control of steroid hormones) and the full maturation of the brain and its neuronal rearrangements. All these changes are an important source of stress and can promote long-lasting vulnerability or resilience processes, including a higher risk of experiencing psychotropic substances. The systematic review by Sicher et al. provides a composite picture of the interplay between the different neural, hormonal, and environmental actors involved in cortical development during adolescence, and of their interaction with biological sex, stress, and alcohol. Important limits of the current literature are also highlighted, i.e., the presence of crucial differences in the protocols applied in laboratory rodent studies and the need to better characterize the complexity of these interactions in human studies by considering not only the biological sex but also the gender identity and the great variability in the patterns of alcohol consumption that characterize adolescents.

Using a voluntary drinking paradigm in mice, Floris et al. investigated alcohol consumption and preference in animals lacking functional GABA-B receptors. Interestingly, they found a higher alcohol intake in fully transgenic mice than in wild-type controls, which seems to be linked to conformation changes involving the δ subunit in GABA-A receptors at the level of the hippocampus. These results confirm a functional connection between the two GABA receptors and suggest that alterations in their crosstalk in specific brain areas are associated with increased alcohol consumption. The discovery that GABA-B receptor deficiency resulted in a lack of alcohol-induced neurosteroid expression in the hippocampus further supports the functional interactions between GABA receptors and neurosteroids in alcohol consumption.

With the aim to investigate the neurophysiological mechanisms underlying the pathological interaction between prenatal infections and the developing brain, Santoni et al. examined the transgenerational effects of maternal immune activation (MIA) on the mesolimbic dopamine pathway of the second generation (F2) of male and female rats. By combining behavioral and electrophysiological characterization of the dopamine system in the MIA model across generations, the authors showed that maternal infections during pregnancy in female rats of the first (F0) generation influence in a sex-dependent manner the dopamine system integrity not only in their offspring (F1) but also in the F2 progeny, confirming that MIA influences the neurodevelopmental trajectories in the dopamine function across generations.

To shed light on the mechanisms underlying the long-lasting effects of early life stress on the brain, Chen et al. investigated the effects of maternal separation on the synaptic excitatory/inhibitory (E/I) balance in the medial prefrontal cortex during adolescence and adulthood. By performing behavioral tests, whole-cell recordings, and intracerebral microdialysis coupled with HPLC-MS in rats, the authors provided evidence that different behavioral age-dependent deficits caused by maternal separation and observed in both adolescence and adulthood may be due to an E/I imbalance as a result of presynaptic glutamate release alterations.

The last contribution to this Research Topic focused on two related peptides, arginine vasopressin (AVP) and oxytocin (OT), which are associated with a broad range of physiological actions both at the central and peripheral level, including parturition. The original research by Malfertheiner et al. shows that birth stress is associated more strictly with the activity of AVP and copeptin (the C-terminal part of AVP prohormone) than with that of OT, which is unaffected by the specific birth stress conditions (Caesarean section vs. vaginal delivery). Due to their pervasive roles in the regulation of social behaviors and stress responses, the effects of these hormones in early life experiences deserve further investigation also to better understand their long-lasting neurobiological and behavioral outcomes.

## Author contributions

All authors listed have made a substantial, direct, and intellectual contribution to the work and approved it for publication.

## Conflict of interest

The authors declare that the research was conducted in the absence of any commercial or financial relationships that could be construed as a potential conflict of interest.

## Publisher's note

All claims expressed in this article are solely those of the authors and do not necessarily represent those of their affiliated organizations, or those of the publisher, the editors and the reviewers. Any product that may be evaluated in this article, or claim that may be made by its manufacturer, is not guaranteed or endorsed by the publisher.
